# Ferredoxin Gene Mutation in Iranian *Trichomonas vaginalis* Isolates

**Published:** 2013

**Authors:** Soudabeh HEIDARI, Mojgan BANDEHPOUR, Seyyed-Javad SEYYED-TABAEI, Zarintaj VALADKHANI, Ali HAGHIGHI, AliReza ABADI, Bahram KAZEMI

**Affiliations:** 1 Parasitology and Mycology Dept. Shahid Beheshti University of Medical Sciences, Tehran, Iran; 2 Cellular and Molecular Biology Research Center, Shahid Beheshti University of Medical Sciences, Tehran, Iran; 3 Biotechnology Dept. Shahid Beheshti University of Medical Sciences, Tehran, Iran; 4 Parasitology Dept. Pasteur Institute of Iran, Tehran, Iran; 5 Social Medicine Dept. Shahid Beheshti University of Medical Sciences, Tehran, Iran

**Keywords:** *Trichomonas vaginalis*, Mutation, Ferredoxin gene

## Abstract

**Background:**

*Trichomonas vaginalis* causes trichomoniasis and metronidazole is its chosen drug for treatment. Ferredoxin has role in electron transport and carbohydrate metabolism and the conversion of an inactive form of metronidazole (CO) to its active form (CPR). Ferredoxin gene mutations reduce gene expression and increase its resistance to metronidazole. In this study, the frequency of ferredoxin gene mutations in clinical isolates of *T.vaginalis* in Tehran has been studied.

**Methods:**

Forty six clinical *T. vaginalis* isolates of vaginal secretions and urine sediment were collected from Tehran Province since 2011 till 2012. DNA was extracted and ferredoxin gene was amplified by PCR technique. The ferredoxin gene PCR products were sequenced to determine gene mutations.

**Results:**

In four isolates (8.69%) point mutation at nucleotide position -239 (the translation start codon) of the ferredoxin gene were detected in which adenosine were converted to thymine.

**Conclusion:**

Mutation at nucleotide -239 ferredoxin gene reduces translational regulatory protein's binding affinity which concludes reduction of ferredoxin expression. For this reduction, decrease in activity and decrease in metronidazole drug delivery into the cells occur. Mutations in these four isolates may lead to resistance of them to metronidazole.

## Introduction

*Trichomonas vaginalis*, an extracellular flagell-ated protozoan, causes trichomoniasis, the most common non-viral sexually transmitted disease. It has importance for the medical, social and economic matters. Trichomoniasis’ complications in women include a variety of inflammatory conditions, vaginitis, cervicitis, salpengitis and infertility (1).

*Trichomonas vaginalis* can have adverse effects on pregnancy outcome, rupture of the fetal membranes and by the toxin like secretion causes early labor (before 37^th^ week) and also low birth weight (less than 2500 g), and even fetal death and ectopic pregnancies can also be followed (2). In the case of the impact on the distribution of chromosomes in meiosis type can be considered as the cause of 21st trisomy, and can lead to presence of Down syndrome symptoms in offspring (3). Major fetal complications include nephritis, necrotic enterocolitis, intraventricular hemorrhage, and respiratory failure (4, 5).

Trichomoniasis causes 11-13% Non-gono-coccal urethritis in men (6). This parasite may penetrate into the prostate gland which leads to the prostatitis (7). *Trichomonas vaginalis* metabolites can decrease human sperm motility in in vitro condition and may be a cause of infertility (8). *Trichomonas vaginalis* has a potential role in the development of secondary infection caused by HIV and the human papilloma virus; it also can be responsible for the cervical cancer (9, 10).The chosen drug for trichomoniasis treatment is metronidazole with about 95% cure rate (11). Metronidazole which is in inactive form (oxide) is entered through passive diffusion into *T.vaginalis* and is reduced by pyruvate - Ferredoxin oxidoreductase (PFOR) in hydrogenosome organelle and nitrogen is converted to nitrogen radicals. Electron transfer occurs through ferredoxin between PFOR and metronidazole. Ferredoxin is involved in carbohydrate metabolism (12, 13).

One of the major clinical features of *T.vaginalis* is resistance to metronidazole, the chosen drug for treatment. Metronidazole resistance was reported in 1962 for the first time (14). Although in most cases the treatment with metronidazole have been successful, trichomoniasis metronidazole resistance is increasing. The rate of resistance to metronidazole was reported for 5% in 1989 by CDC (Center for Disease Control) (15). The prevalence of resistance was low till 1996 (one case /year), while it increased up to 17 cases in 1997-1998 in Detorit and Philadelphia (16). In 2001 and 2006, resistance rate was 10% in USA (17) and 9.6% in Birmingham (18) respectively.

The mutations in multiple genes, the lack of a specific protein which reduces the expression of other proteins via metabolic feedback, loss or mutation of a key regulatory molecules such as DNA binding protein which leads to changes in gene expression in hydrogenosome are the mechanisms that due to the drug resistance. It has been suggested that changes in the activity of PFOR and dehydrogenase enzymes lead to reduction of protein expression (19). The reduction of the hydrogenosomal enzymes activity such as PFOR and dehydrogenase were reported by Rasolson et al. which lead to development of drug resistance in in vitro (20).

This is the first study to determine the frequency of Ferredoxin gene mutations in clinical isolates of *T.vaginalis* in Iran.

## Materials and Methods

### Sampling

Forty-six isolates of *T. vaginalis* were collected in Tehran province 2012. Vaginal secretions and urine samples were cultured at 37 °C on the Diamond TYI-S-33 supplemented with 10% fetal calf serum (21), antibiotic (1000 U / mL penicillin, 30 µg / mL streptomycine sulfate) and fungicides (40µg/-mL amphotericin B) were added. Parasites were harvested at stationary phase (2×10^6^ parasites) by centrifugation and storage in -80°C.

### DNA Extraction

Parasites were washed in PBS (pH 7.4) by 8000× g for 10 min at 4 °C and nucleic acids were extracted using nucleic acid extraction kit (Fermentas).

### PCR amplification

PCR reaction was carried out for amplification of *T.vaginalis* ferredoxin gene using specific primers. Specific primers, TV Fer F and TV Fer R, were designed based on Quon et al. report (22). Reverse primer have designed based on ACCESSION number AY-149601. For including the nucleotide position -239 of ferredoxin gene in PCR product, the nine nucleotide (CGACAGATA) was added at 5′ end of forward primer.

TV fer F: 5′ CGACAGATAATAATTTTTTTG-AAAA 3′TV fer R: 5′- TGCAGATGCACTTGC-CGC - 3′PCR reaction was carried out in a 20 microliters final volume including; 1 ng of extracted DNA, 20 picomol of reverse and forward primers, 0.2 mM dNTP, 1.5 unit of Taq DNA polymerase, 1.5 mM MgCl2 and 1X specific PCR buffer. The reaction was performed with the following conditions: denaturation at 94°C for 30 s, primer annealing at 46 ° C for one minute, and primer extension at 72 °C for 30 s, these steps repeated for 40 cycles. PCR product was electrophoresed on 1.5% agarose gel. The mentioned primers were amplified 456-nucleotide from *T.vaginalis* ferredoxin gene.

### Molecular analysis

PCR products were sequenced (by Genetic Analyzer 3730 at Bioneer Company) for detection of point mutation at nucleotide position -239 (relative to the translation start codon) of *T.vaginalis* ferredoxin gene. All sample nucleotide sequences were reviewed and evaluated by BLAST program in GenBank data base.

### Statistical analysis

All information were analyzed using SPSS16 software, the Mann-Whitney statistical analysis showed no significant relationship between age and the presence of mutations in *T. vaginalis* ferredoxin gene (*P*- value > 0.05).

## Results

### Agarose gel electrophoresis of PCR product

**Fig. 1:**
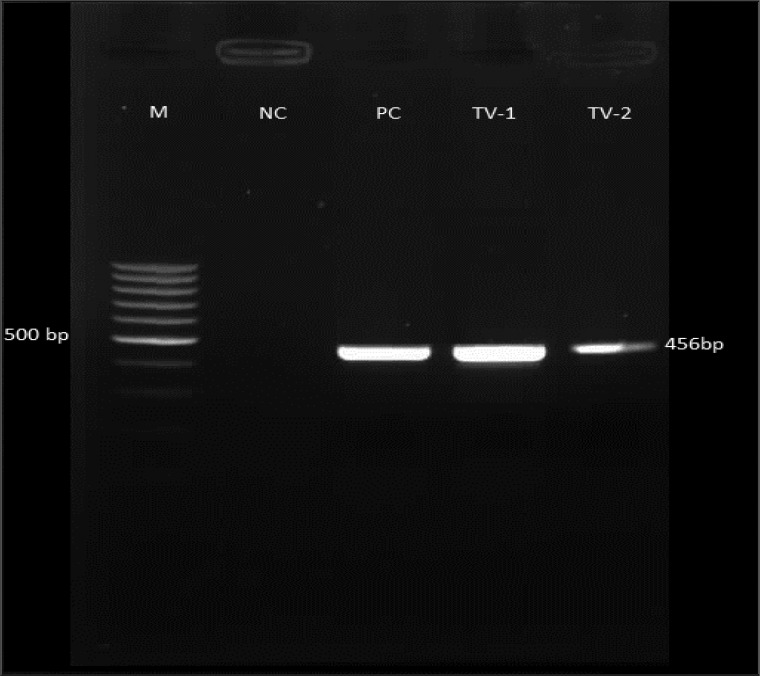
The 1.5% agarose gel electrophoresis. M; 100 bp DNA ladder marker, NC; negative control, PC; positive control, TV; *Trichomonas vaginalis* ferredoxin gene PCR product

In this study, 46 isolates of *T.vaginalis* were analysed by PCR reaction. The 456 bp of ferredoxin gene was amplified in all the isolates. Figure 1 shows 1.5% agarose gel electrophoresis of *T.vaginalis* ferredoxin gene PCR product.

### Ferredoxin gene sequence analysis

**Fig. 2:**
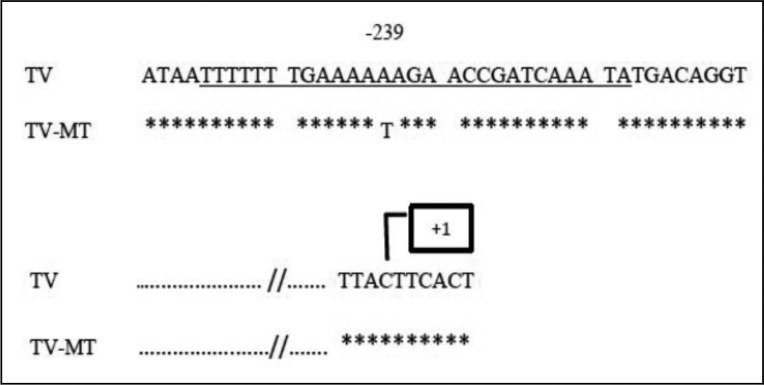
Comparison of the sequence of nucleotides before the translation start codon of the ferredoxin gene by BLAST sofware. TV is nucleotide sequence of wild type isolate and TV-MT is nucleotide sequence of mutant isolate. It shows change in nucleotide position -239, and box shows start of transcription of the ferredoxin gene. The underlined region (28-bp oligomers) is the translational regulatory protein connection zone (Ref 22)

Ferredoxin PCR product of all isolates were purified and sequenced. Sequences were analyzed by BLAST program in GenBank data base. All sample sequences showed 100 to 99% homology with *T. vaginalis* ferredoxin gene. In four isolates (8.69%), a point mutation at nucleotide position -239 was detected in which thymine was replaced by adenine (Fig. 2). Sequences were submited GenBank at accession numbers JQ 969042 (SH 9 for mutant Ferredoxin) and JQ969043 (SH-10 for wild type Ferredoxin).

### Statistical analysis

All information were analyzed using SPSS16 software, the Mann-Whitney statistical analysis showed no significant relationship between age and the presence of mutations in *T.vaginalis* ferredoxin gene (*P-* value > 0.05). The mean of the age of the infected patients with *T.vaginalis* without mutation was 50.75 and the mean of the age of the infected patients with *T.vaginalis* with mutation in ferredoxin gene was 40.60 (Table 1).

**Table 1:** The mean of the age of the patients infected with *T.vaginalis* without mutation and with mutation in ferredoxin gene

Age MTfer	Mean age	Number
**Posetive**	50.75	4
**Negative**	40.60	42
**Total**	41.48	46

## Discussion

The aim of this study was to determine *T. vaginalis* ferredoxin gene mutation in clinical isolates in Tehran. Previous studies had shown a strong correlation between the decrease in intracellular ferredoxin and drug resistance (22). Reduction in the amount of ferredoxin gene translation and mRNA in resistant strains against the susceptible strains (22) raises this hypothesis that by the decrease of the cells’ ability to regenerate metronidazole into cytotoxic forms, the drug resistance occurs. Decrease in gene transcription may be due to the mutations in the nucleotide position –239 which lead to a decrease in 23 kDa regulatory protein binding affinity and a decrease in gene expression in ferredoxin. We reported the 8.69% mutant strains in this study.In previous studies the mutation was found only in some resistant isolates (22). The presence of unidentified mutations in ferredoxin gene and also the presence of mutations in activating transcription factors (transacting factors) could be other reasons for the reduction of ferredoxin gene translation in other resistant isolates (22). Qoun et al. reported that the ferredoxin expression reduction was because of point mutation at position -239 in the ferredoxin gene (22). There is not absolute function of ferredoxin in metronidazole resistance in *T.vaginalis*, some studies proposed involvement of ferredoxin in sensitivity to nitroimidazole (23) while other findings showed that the inhibition of ferredoxin gene in in vitro does not lead to metronidazole resistance (24). There is no study about frequency of mutation in ferredoxin gene in Iran. Further in vitro and in vivo studies are needed to evaluate the relationship between ferredoxin gene mutation and its resistance to metronidazole in clinical isolates of *T.vaginalis*.

## Conclusion

In this study we detected a point mutation at nucleotide position -239 of ferredoxin gene in 8.69% of Iranian *Trichomonas vaginalis* isolates.
